# Novel lipid indicators and the risk of type 2 diabetes mellitus among Chinese hypertensive patients: findings from the Guangzhou Heart Study

**DOI:** 10.1186/s12933-022-01660-z

**Published:** 2022-10-15

**Authors:** Hai Deng, Peng Hu, Huoxing Li, Huanning Zhou, Xiuyi Wu, Maohua Yuan, Xueru Duan, Miaochao Lao, Chuchu Wu, Murui Zheng, Xiang Qian Lao, Wenjing Zhao, Xudong Liu

**Affiliations:** 1 Department of Cardiology, Guangdong Cardiovascular Institute, Guangdong Provincial People’s Hospital, Guangdong Academy of Medical Science, Guangzhou, 510080 China; 2 School of Public Health, Guangdong Pharmaceutical University, No. 283 Jianghai Avenue, Haizhu District, Guangzhou, 510310 China; 3 Department of Epidemiology, School of Public Health, Sun Yat-Sen University, Guangzhou, 510080 China; 4 The Second School of Clinical Medicine, Southern Medical University, Guangzhou, 510515 China; 5 Guangzhou Yuexiu District Center for Disease Control and Prevention, Guangzhou, China; 6 Nancun Community Health Service Center, Guangzhou, 511442 China; 7 Dadong Street Community Health Service Center, Guangzhou, 510080 China; 8 Department of Sleep Center, Department of Geriatric Respiratory, Guangdong Provincial People’s Hospital, Guangdong Academy of Medical Sciences, Guangdong Provincial Geriatrics Institute, Guangzhou, 510080 China; 9 Department of Community Health, Guangzhou Center for Disease Control and Prevention, No. 1 Qide Road, Baiyun District, Guangzhou, 510440 China; 10 JC School of Public Health and Primary Care, Faculty of Medicine, The Chinese University of Hong Kong, Hong Kong, 999077 SAR China; 11 School of Public Health and Emergency Management, Southern University of Science and Technology, No. 1088 Xueyuan Avenue, Shenzhen, 518055 China

**Keywords:** Hypertriglyceridemic-waist phenotype, Triglyceride glucose index, Lipid accumulation product, Visceral adiposity index, Diabetes, Hypertension

## Abstract

**Background:**

Data are limited on whether several easily measured indices are independent predictors of type 2 diabetes mellitus (T2DM) in hypertensive patients. This study aimed to assess the association of hypertriglyceridemic-waist phenotype, triglyceride glucose (TyG) index, lipid accumulation product (LAP), and visceral adiposity index (VAI) with T2DM risk in hypertensive patients.

**Methods:**

This cross-sectional study included 5321 hypertensive patients from the baseline survey of the Guangzhou Heart Study. Face-to-face questionnaire survey, physical examination, and fasting blood sample collection were completed for all subjects. Odds ratio (OR) with 95% confidence interval (95% CI) were calculated by using the logistic regression model. The potential nonlinear relationship was examined using restricted cubic spline regression.

**Results:**

The prevalence of T2DM was 19.98% among hypertensive patients. After adjusting for confounders, participants with elevated triglyceride levels and enlarged waist circumference (HTGW) were associated with a 2.57-fold risk of T2DM (OR 2.57, 95% CI 2.05, 3.23). When comparing with subjects within the lowest quartile of the indices, those in the highest quartile of TyG, LAP, and VAI were associated with 5.35-fold (95% CI 4.33, 6.64), 2.65-fold (95% CI 2.11, 3.34), and 2.17-fold (95% CI 1.77, 2.67) risk of T2DM after adjusting for confounders. Every 1-unit increment of TyG, LAP, and VAI was associated with 81%, 38%, and 31% increased risk of T2DM, respectively. The nonlinear association was observed for TyG, LAP, and VAI (all *P*_Non-linear_ < 0.001).

**Conclusions:**

The results found that among hypertensive patients, HTGW and a higher level of TyG, LAP, and VAI were associated with an elevated risk of T2DM. The findings suggested that HTGW, TyG, LAP, and VAI may serve as simple and effective tools for T2DM risk assessment in the prevention and management of main chronic diseases.

**Supplementary Information:**

The online version contains supplementary material available at 10.1186/s12933-022-01660-z.

## Background

Type 2 diabetes mellitus (T2DM) has been found to be associated with an increased risk of cardiovascular diseases and reduced life expectancy, imposing a huge economic burden on societies [1–3]. The International Diabetes Federation estimated that the number of individuals with diabetes will be 700 million in 2045, and 80% will be found in low- and middle-income countries [4]. China has the largest number of diabetics in the world, with nearly 140.9 million diabetics in 2021 [5, 6]. With lifestyle changes and nutritional transitions over the past decades, diabetes has become a serious public health problem in China [5, 6].

Visceral adiposity and insulin resistance are associated with an increased risk of diabetes [7]. Imaging techniques, such as computed tomography and magnetic resonance imaging, can measure fat distribution and evaluate visceral adiposity quantitatively [8]; the hyperinsulinemic-euglycemic clamp is the gold standard test for the measurement of insulin resistance [9]. However, these techniques are time-consuming and costly for regular medical examinations and large-scale epidemiological studies. Thus, a simple and cost-effective predictor is required for detecting subjects with a high risk of T2DM.

Some anthropometric measures were usually used to evaluate the risk of cardiovascular diseases and metabolic syndrome [10–14]. By combining these anthropometric measures with biochemical measures, some novel lipid indicators can be established. The hypertriglyceridemic-waist phenotype, defined as an elevated triglyceride (TG) level and enlarged waist circumference (WC), was firstly introduced in 2000 as a useful and easy-to-measure marker of excess visceral adiposity [15]. In addition, the triglyceride glucose (TyG) index, the product of TG and fasting plasma glucose (FPG), has been considered as a surrogate measure to identify insulin resistance and define the status of metabolic health [16, 17]. Moreover, another two inexpensive and reliable lipid indices have been proposed to estimate insulin resistance from visceral adiposity dysfunction [18, 19]. One is lipid accumulation product (LAP), a model calculated from WC and TG levels [18]; the other is visceral adiposity index (VAI), which is computed based on body mass index (BMI), WC, TG, and high-density lipoprotein cholesterol (HDL-C) [19].

Prior studies have indicated that elevated triglyceride level and enlarged waist circumference (HTGW) was risk factor for developing T2DM [20–23]. Several studies have also demonstrated that higher levels of TyG, LAP, and VAI indices were associated with an increased risk of T2DM [24–26]. However, most of the previous studies were conducted among the general population. Compared with normotensive subjects, diabetes mellitus is more frequent in hypertensive patients [27, 28]. Hypertension could induce endothelial dysfunction, increase inflammatory markers, and further lead to the incidence of diabetes [29, 30]. Hence, hypertension may be a provoking factor for developing diabetes. However, whether these lipid indices have similar predictive effects on T2DM among hypertension patients is still unclear. Therefore, this population-based study was performed to evaluate the association between HTGW, TyG, LAP, and VAI indices and the risk of T2DM in subjects with hypertension, and to examine any possible effect modifiers.

## Methods

### Setting and subjects

The data used in this population-based cross-sectional study was derived from the baseline survey of Guangzhou Heart Study, an ongoing prospective cohort study in Guangzhou, China. Detailed information about Guangzhou Heart Study has been shown in our previous reports [31–35]. Briefly, a total of 12,013 permanent residents aged 35 years or above were recruited and accomplished the baseline survey between July 2015 and August 2017. Those who had mental or cognitive disorders, had mobility difficulties, had any history of cancer, were pregnant or lactating women, and were non-Guangzhou permanent residents were excluded when recruiting subjects. In this study, we further excluded those with missing data on the blood pressure measurement or diabetes-related information (n = 6), and subjects without hypertension (n = 6686). Finally, a total of 5321 subjects with hypertension were included for further analysis. No significant difference in terms of age and sex was observed between subjects included and not included. The flow chart of the selection of hypertensive patients were shown in Additional file 1: Fig. S1.

This study was approved by the Ethical Review Committee for Biomedical Research, School of Public Health, Sun Yat-sen University, and the Research Ethics Committee for Guangdong Provincial People’s Hospita1, Guangdong Academy of Medical Sciences. The study was performed in line with the Declaration of Helsinki and all participants provided informed consent.

### Data collection and measurements

A structured questionnaire conducted with a face-to-face approach was used to collect demographic and lifestyle information, including age (years), sex (male, female), education (< high school, ≥ high school), marital status (married, others), retirement status (yes, no), smoking (yes, no), and alcohol drinking (yes: occasion and frequent, no: never drinking). Leisure-time physical activity was estimated by using the modified Global Physical Activity Questionnaire (GPAQ) with in-person interview [33, 34]. The volume of each type of leisure-time physical activity was calculated by multiplying the intensity of the physical activity by its frequency and its duration, and the total volume of leisure-time physical activity (MET-h/week) was the sum of the volume of all types of leisure-time physical activity [33, 34]. Medical history including physician-diagnosed T2DM (yes, no), hypertension (yes, no), and cardiovascular diseases (yes, no) was required to report.

Physical measurements, including height, weight, waist circumference (WC), and blood pressure, were performed by trained medical workers in line with standard instruments and protocols. BMI was calculated by dividing weight by height squared (kg/m^2^). WC was measured with a tape at the midway between the lower edge of the costal arch and the superior border of the iliac crest while standing. Participants were instructed to rest for at least 10 min in a quiet room before blood pressure measurement. Systolic blood pressure (SBP) and diastolic blood pressure (DBP) were measured three times by trained medical workers, and then the mean of three measurements was calculated. Hypertension was defined as SBP ≥ 140 mmHg or DBP ≥ 90 mmHg, or having self-reported physician-diagnosed hypertension [33].

Fasting blood sample from each subject was collected, and serum concentrations of FPG, TG, HDL-C, low-density lipoprotein cholesterol (LDL-C), and total cholesterol were detected. T2DM was defined as fasting blood glucose ≥ 7.0 mmol/L, or having self-reported physician-diagnosed diabetes excluding gestational diabetes mellitus, type 1 diabetes mellitus, or other types of diabetes [36].

### Definition of indicators

Central obesity was defined as a WC ≥ 85 cm in women or ≥ 90 cm in men [37]. TG level ≥ 1.7 mmol/L was defined as increased TG [38]. The hypertriglyceridemic-waist phenotypes were categorized into four groups: (1) normal triglyceride level and normal waist circumference (NTNW); (2) normal triglyceride level and enlarged waist circumference (NTGW); (3) elevated triglyceride level and normal waist circumference (HTNW); (4) elevated triglyceride level and enlarged waist circumference (HTGW). TyG, LAP and, VAI were calculated using the following formulas [39]:

$$\mathrm{TyG}=\mathrm{ln}[\mathrm{TG}\left(\frac{\mathrm{mg}}{\mathrm{dL}}\right)\times \mathrm{FPG}(\mathrm{mg}/\mathrm{dL})/2].$$

$$\mathrm{LAP}\left(\mathrm{males}\right)=\left[\mathrm{WC}\left(\mathrm{cm}\right)-65\right]\times \mathrm{TG}\left(\frac{\mathrm{mmol}}{\mathrm{L}}\right).$$

$$\mathrm{LAP}\left(\mathrm{females}\right)=\left[\mathrm{WC}\left(\mathrm{cm}\right)-58\right]\times \mathrm{TG}\left(\frac{\mathrm{mmol}}{\mathrm{L}}\right).$$

$$\mathrm{VAI}\left(\mathrm{males}\right)=\left[\frac{\mathrm{WC}\left(\mathrm{cm}\right)}{39.68+1.88\times \mathrm{BMI}(\mathrm{kg}/{m}^{2})}\right]\times \left[\frac{\mathrm{TG}\left(\mathrm{mmol}/\mathrm{L}\right)}{1.03}\right]\times \left[\frac{1.31}{\mathrm{HDL}-\mathrm{C}\left(\frac{\mathrm{mmol}}{\mathrm{L}}\right)}\right].$$

$$\mathrm{VAI}\left(\mathrm{females}\right)=\left[\frac{\mathrm{WC}\left(\mathrm{cm}\right)}{36.58+1.89\times \mathrm{BMI}(\mathrm{kg}/{m}^{2})}\right]\times \left[\frac{\mathrm{TG}\left(\mathrm{mmol}/\mathrm{L}\right)}{0.81}\right]\times \left[\frac{1.52}{\mathrm{HDL}-\mathrm{C}\left(\frac{\mathrm{mmol}}{\mathrm{L}}\right)}\right].$$

### Statistical analysis

The normality was examined by the Kolmogorov–Smirnov test. The continuous variables with normal distribution were displayed using mean and standard deviation (SD), and the continuous variables with non-normal distribution were shown using median and interquartile range (IQR). The distribution of categorical variables was represented as frequency and proportion (%). The distribution difference of baseline demographics, lifestyle information, and other covariables among different groups was estimated by chi-square test for a categorical variable and by t-test or Wilcoxon rank-sum test for a continuous variable. The continuous variables of TyG, LAP, and VAI were transformed into categorical variables by using quartile methods.

Logistic regression models were performed to estimate the association of T2DM risk with triglyceridemic-waist phenotypes, TyG, LAP, and VAI; odds ratios (ORs) with 95% confidence intervals (CIs) were calculated with and without adjustment for covariates. The potential nonlinear relationship was examined using restricted cubic spline regression (knots on the 5th, 25th, 75th, and 95th percentiles), with OR and 95% CI being calculated based on the logistic regression model. The restricted cubic spline regression was performed with the “rms” package.

Stratified analysis was conducted by age group (< 60 or ≥ 60 years), sex (male or female), retirement status (retirement or non-retirement), education (< High school or ≥ High school), BMI (< 24 or ≥ 24 kg/m^2^), smoking (yes or no), and alcohol drinking (yes or no). The multiplicative interaction of HTGW, TyG, LAP, and VAI with age group, sex, retirement status, education, BMI, smoking, and alcohol drinking was estimated respectively using the likelihood ratio test, with a comparison of the likelihood scores of the two models with and without the interaction terms. In addition, to examine the robustness of the results, we conducted the sensitivity analyses by defining WC ≥ 80 cm in women as enlarged waist circumference according to the new International Diabetes Federation; and by adding total cholesterol and LDL-C as additional covariates to control potential influence; and by defining hypertension as SBP ≥ 130 mmHg or DBP ≥ 85 mmHg, or having self-reported physician-diagnosed hypertension. All statistical analyses were performed using R 4.0.1 (R Development Core Team, Vienna, Austria); the tests were two-tailed, and the *P* value of less than 0.05 was considered statistically significant.

## Results

A total of 5,321 subjects with hypertension were included in the present study, of which 4,258 subjects (80.02%) were classified into the non-T2DM group and 1,063 (19.98%) into the T2DM group (Table 1). Of all subjects, the mean (SD) of age, BMI, SBP, DBP and TyG index was 64.08 (10.72) years, 24.96 (3.61) kg/m^2^, 142.70 (16.79) mmHg, 85.71 (11.19) mmHg, and 8.82 (0.63). The median (IQR) value of the leisure-time physical activity, LAP, and VAI was 34.65 (39.90) MET-h/week, 38.92 (38.07), and 1.74 (1.85). More participants in the T2DM group than in the non-T2DM group were non-married, retirees, and non-alcohol drinker, had a history of cardiovascular diseases, were in the HTGW group, and were in the highest quartile of TyG, LAP and VAI (all *P* < 0.05).

**Table 1:** Baseline characteristic of the subjects with hypertension

Characteristic	Total (N = 5321)	Non-T2DM (N = 4258)	T2DM (N = 1063)	*P*
Age (years), mean (S.D.)	64.08 (10.72)	63.44 (10.79)	66.66 (10.00)	< 0.001^*^
BMI (kg/m^2^), mean (S.D.)	24.96 (3.61)	24.85 (3.59)	25.41 (3.67)	< 0.001^*^
Leisure-time physical activity (MET-h), median (Interquartile)	34.65 (39.90)	34.65 (40.79)	34.65 (38.91)	0.631^†^
SBP (mmHg), mean (S.D.)	142.70 (16.79)	142.80 (16.67)	142.30 (17.29)	0.340^*^
DBP (mmHg), mean (S.D.)	85.71 (11.19)	86.45 (11.03)	82.74 (11.30)	< 0.001^*^
TyG index, mean (S.D.)	8.82 (0.63)	8.73 (0.55)	9.19 (0.76)	< 0.001^*^
LAP, median (interquartile)	38.92 (38.07)	37.27 (35.63)	47.92 (46.61)	< 0.001^†^
VAI, median (interquartile)	1.74 (1.85)	1.65 (1.73)	2.14 (2.32)	< 0.001^†^
Sex, N (%)				0.909^‡^
Male	2078 (39.05)	1665 (39.10)	413 (38.85)	
Female	3243 (60.95)	2593 (60.90)	650 (61.15)	
Education, N (%)				0.540^‡^
< high school	3668 (68.93)	2944 (69.14)	724 (68.11)	
≥ high school	1653 (31.07)	1314 (30.86)	339 (31.89)	
Material status, N (%)				0.029^‡^
Married	4331 (81.39)	3491 (81.99)	840 (79.02)	
Others	990 (18.61)	767 (18.01)	223 (20.98)	
Retirement status, N (%)				< 0.001^‡^
Non-retirement	1555 (29.22)	1339 (31.45)	216 (20.32)	
Retirement	3766 (70.78)	2919 (68.55)	847 (79.68)	
Smoking, N (%)				0.252^‡^
No	4107 (77.18)	3272 (76.84)	835 (78.55)	
Yes	1214 (22.82)	986 (23.16)	228 (21.45)	
Alcohol drinking, N (%)				0.021^‡^
No	4252 (79.91)	3375 (79.26)	877 (82.50)	
Yes	1069 (20.09)	883 (20.74)	186 (17.50)	
History of cardiovascular diseases, N (%)				< 0.001^‡^
No	4951 (93.05)	3989 (93.68)	962 (90.50)	
Yes	370 (6.95)	269 (6.32)	101 (9.50)	
Triglyceridemic-waist phenotypes, N (%)				< 0.001^‡^
NTNW	1628 (30.60)	1412 (33.16)	216 (20.32)	
NTGW	1652 (31.05)	1309 (30.74)	343 (32.27)	
HTNW	774 (14.55)	622 (14.61)	152 (14.30)	
HTGW	1267 (23.81)	915 (21.49)	352 (33.11)	
TyG (quartile), N (%)				< 0.001^‡^
Quartile 1 (≤ 8.39)	1330 (25.00)	1185 (27.83)	145 (13.64)	
Quartile 2 (8.39–8.76)	1330 (25.00)	1153 (27.08)	177 (16.65)	
Quartile 3 (8.76–9.18)	1331 (25.00)	1088 (25.55)	243 (22.86)	
Quartile 4 (> 9.18)	1330 (25.00)	832 (19.54)	498 (46.85)	
LAP (quartile), N (%)				< 0.001^‡^
Quartile 1 (≤ 24.31)	1330 (25.00)	1151 (27.00)	179 (16.80)	
Quartile 2 (24.31–38.92)	1330 (25.00)	1097 (25.80)	233 (21.90)	
Quartile 3 (38.92–62.38)	1330 (25.00)	1050 (24.70)	280 (26.30)	
Quartile 4 (> 62.38)	1331 (25.00)	960 (22.50)	371 (34.90)	
VAI (quartile), N (%)				< 0.001^‡^
Quartile 1 (≤ 1.05)	1330 (25.00)	1124 (26.40)	206 (19.40)	
Quartile 2 (1.05–1.74)	1330 (25.00)	1113 (26.10)	217 (20.40)	
Quartile 3 (1.74–2.90)	1331 (25.00)	1052 (24.70)	279 (26.20)	
Quartile 4 (> 2.90)	1330 (25.00)	969 (22.80)	361 (34.00)	

*BMI* body mass index, *HTGW* elevated triglyceride level and enlarged waist circumference, *HTNW* elevated triglyceride level and normal waist circumference, *LAP* lipid accumulation product, *MET-h* metabolic equivalent values-hours, *NTGW* normal triglyceride level and enlarged waist circumference, *NTNW* normal triglyceride level and normal waist circumference, *TyG* triglyceride-glucose, *VAI* visceral adiposity index, *SBP* systolic blood pressure, *DBP* diastolic blood pressure

^*^P value from *t*-test

^†^*P* value from Wilcoxon rank sum test

^‡^*P* value Chi-square test

**Fig. 1:**
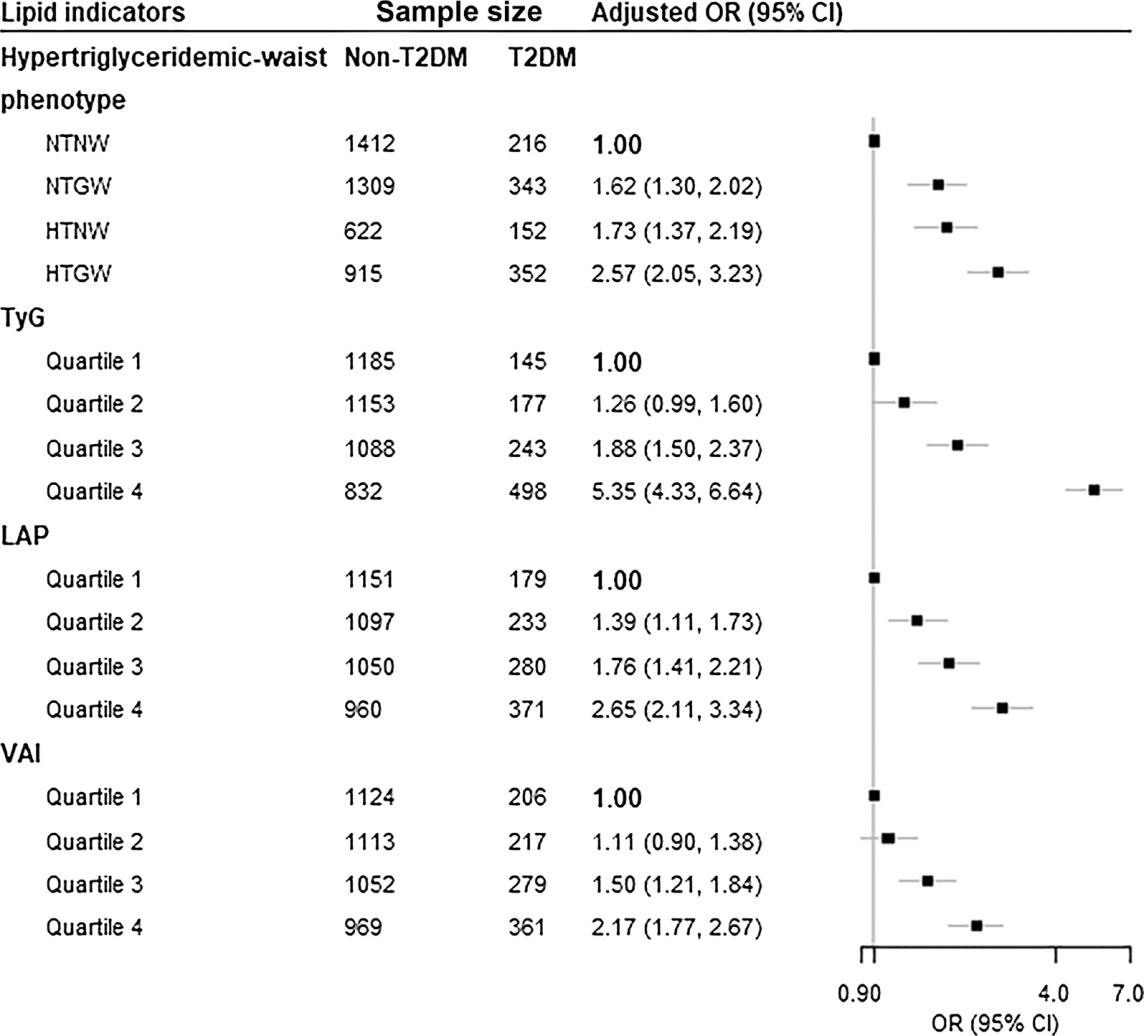
Association between lipid indicators and the risk of T2DM. *HTGW* elevated triglyceride level and enlarged waist circumference, *HTNW* elevated triglyceride level and normal waist circumference; LAP, lipid accumulation product, *NTGW* normal triglyceride level and enlarged waist circumference, *NTNW* normal triglyceride level and normal waist circumference, *TyG* triglyceride-glucose, *VAI* visceral adiposity index. Adjustment for age, sex, education, marital status, retirement status, smoking, alcohol drinking, leisure-time physical activity, body mass index, systolic blood pressure, diastolic blood pressure, and history of cardiovascular disease

The association of different hypertriglyceridemic-waist phenotypes and T2DM risk was shown in Fig. 1 and Additional file 1: Table S1. In comparison to participants with NTNW, those with NTGW, HTNW and HTGW were associated with a 1.62-fold (OR 1.62, 95% CI 1.30, 2.02), 1.73-fold (OR 1.73, 95% CI 1.37, 2.19), and 2.57-fold (OR 2.57, 95% CI 2.05, 3.23) risk of T2DM, respectively, after adjusting for confounders.

**Fig. 2:**
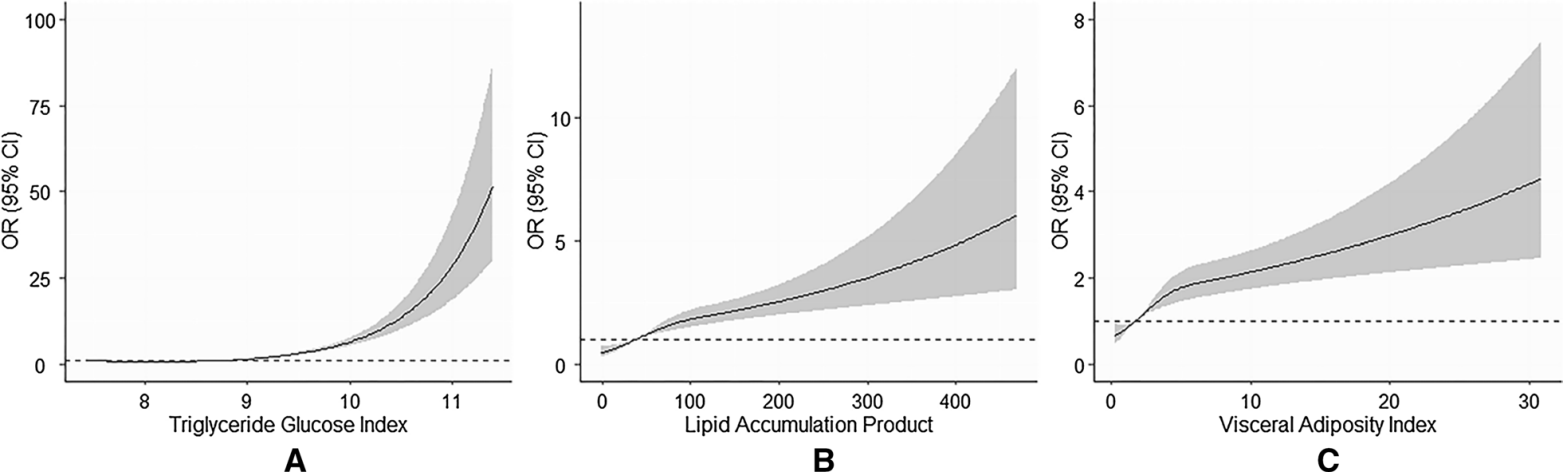
Restricted cubic spline of the relationship of T2DM with TyG (**A**), LAP (**B**), and VAI (**C**). Potential nonlinear relationships were examined using restricted cubic splines (knots on the 5th, 25th, 75th and 95th percentiles), with hazard ratios (HRs) based on logistic regression models. The dash line represents the OR equal to 1. The ORs was adjusted for age, sex, education, marital status, retirement status, smoking, alcohol drinking, leisure-time physical activity, BMI, SBP, DBP and history of cardiovascular diseases

The risk of T2DM increased with the increment of TyG, LAP, and VAI (Fig. 1; Additional file 1: Table S1). After considering possible confounders, every 1-unit increment of TyG, LAP, and VAI were associated with 81% (OR 1.81, 95% CI 1.69, 1.94), 38% (OR 1.38, 95% CI 1.28, 1.48), and 31% (OR 1.31, 95% CI 1.23, 1.40) increased risk of T2DM, respectively. When comparing with the lowest quartile of the indices, the highest quartile of TyG, LAP, and VAI were associated with 5.35-fold (95% CI 4.33, 6.64, *P*_-trend_ < 0.001), 2.65-fold (95% CI 2.11, 3.34, *P*_-trend_ < 0.001), and 2.17-fold (95% CI 1.77, 2.67, *P*_-trend_ < 0.001) risk of T2DM after adjusting for potential confounders. Figure 2 displayed the associations of T2DM risk with TyG, LAP, and VAI through the restricted cubic spline curve, and the significant non-linear associations were found (all *P*_Non-linear_ < 0.001).

**Fig. 3:**
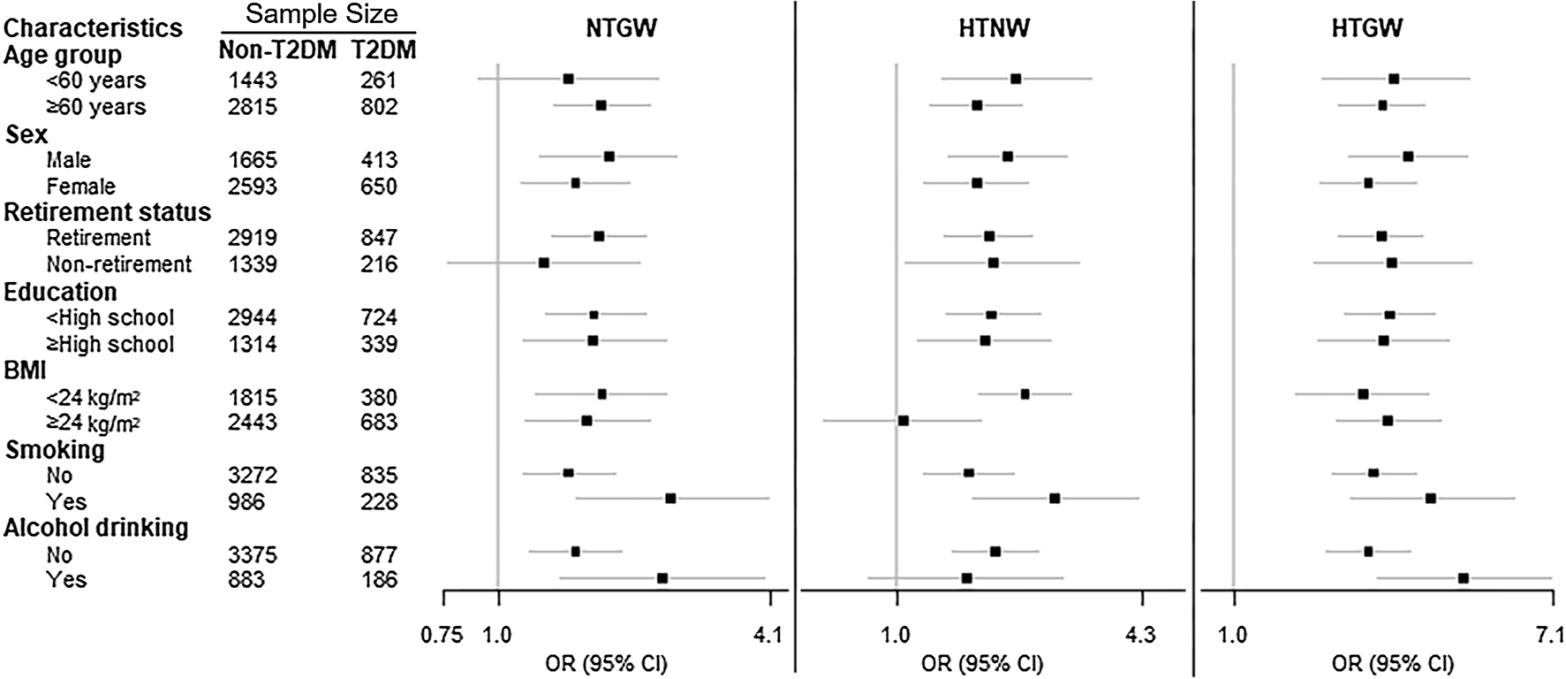
Stratified analyses on the association between triglyceridemic-waist phenotypes and T2DM risk. *BMI* body mass index, *HTGW* elevated triglyceride level and enlarged waist circumference, *HTNW* elevated triglyceride level and normal waist circumference, *NTGW* normal triglyceride level and enlarged waist circumference, *NTNW* normal triglyceride level and normal waist circumference. Adjustment for age, sex, education, marital status, retirement status, smoking, alcohol drinking, leisure-time physical activity, BMI, SBP, DBP and history of cardiovascular diseases except the corresponding stratification variable

Compared with the NTNW group, the HTGW group was significantly associated with an increased risk of T2DM in all subgroups (Fig. 3; Additional file 1: Table S2). Compared with the NTNW group, participants with NTGW were significantly associated an increased risk of T2DM in all stratified groups except for non-retirees and those aged < 60 years, participants with HTNW were significant associated with an increased risk of T2DM in all stratified groups except for alcohol drinkers and those with BMI ≥ 24 kg/m^2^. We also found that BMI was a significant effect modifier in the association between hypertriglyceridemic-waist phenotype and T2DM risk among hypertensive patients (*P*_-interaction_ = 0.008), while no interaction was found between other factors and hypertriglyceridemic-waist phenotype.

**Fig. 4:**
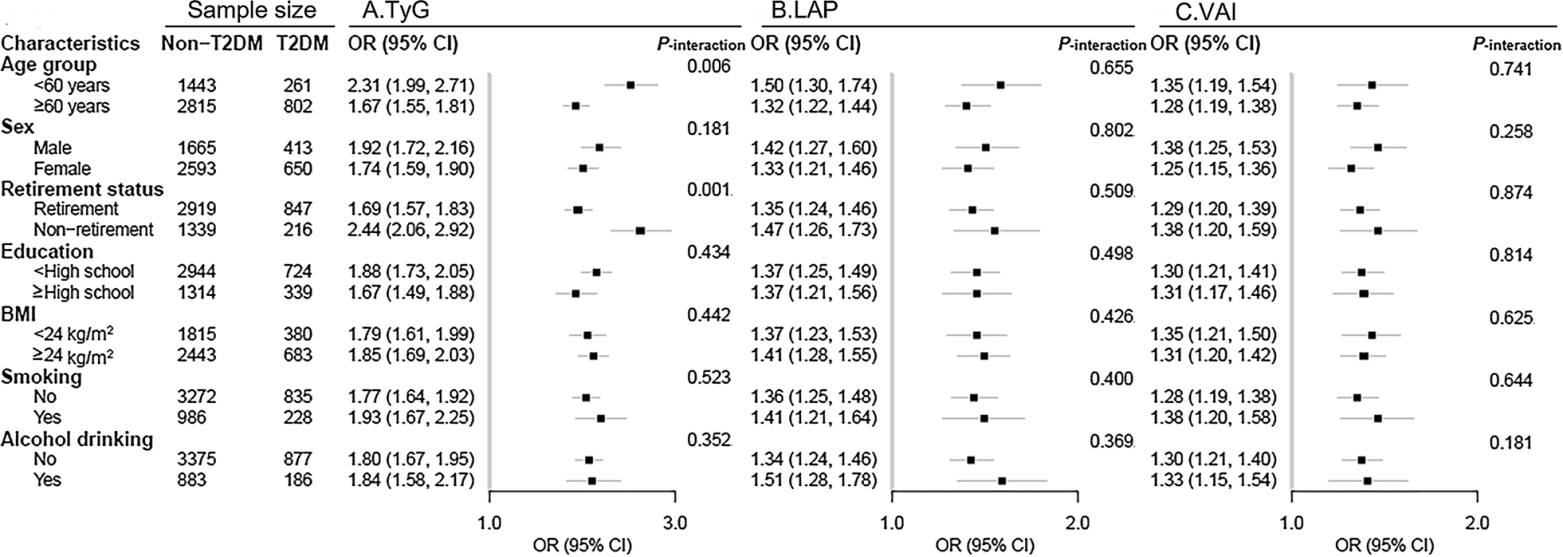
Stratified analyses on the association between TyG, LAP, and VAI and T2DM risk. *BMI* body mass index, *TyG* triglyceride-glucose, *VAI* visceral adiposity index, *LAP* lipid accumulation product. Adjustment for age, sex, education, marital status, retirement status, smoking, alcohol drinking, leisure-time physical activity, BMI, SBP, DBP and history of cardiovascular diseases except the corresponding stratification variable

In stratified analysis, the positive relationship of TyG, LAP, and VAI with T2DM risk remained significant in all subgroups (Fig. 4; Additional file 1: Tables S3–S5). The association between TyG index and T2DM risk appeared stronger in subjects < 60 years and in non-retirees (both *P*_-interaction_ < 0.05). No interaction was found for other factors with TyG, LAP, and VAI.

In sensitivity analysis, similar results on the association between HTGW phenotype and T2DM risk were found when defining WC ≥ 80 cm in women as enlarged waist circumference (Additional file 1: Table S6). In addition, additional adjustment for total cholesterol and LDL-C did not alter the results remarkably (Additional file 1: Table S7); and similar results were also found by using different definition of hypertension (Additional file 1: Table S8).

## Discussion

The present study demonstrated that among hypertensive patients, HTGW was associated with an increased T2DM risk, and TyG, LAP, and VAI were positively associated with the T2DM risk. Furthermore, our study indicated that HTGW was a stable risk factor for T2DM in hypertension patients regardless of any characteristics; and the positive association of TyG index with T2DM was more pronounced in participants < 60 years and in non-retirees.

The hypertriglyceridemic-waist phenotype, defined as elevated TG and enlarged WC, was a simple marker of visceral adiposity [15]. Previous studies indicated that HTGW was associated with higher risk of diabetes, hypertension, and cardiovascular diseases [20–22, 40, 41]. A meta-analysis further indicated that HTGW was closely associated with an increased risk of diabetes in the general population [20]. In the present study, we further found that HTGW was associated with a higher risk of diabetes among hypertensive patients, which is consistent with the results in the general population [20]. Our study showed that the risk effect of HTNW on T2DM was better than subjects with NTGW, which is consistent with several studies [42, 43]; however, some studies indicated that NTGW had a stronger association with T2DM than NTGW [21, 23]. The discrepancies among the studies may be due to the heterogeneity of study designs and study populations. Meanwhile, we found that HTGW was associated with a higher diabetes risk than solely-increased TG or solely-increased WC, which further supported previous studies pointing that participants’ cardiovascular disease risk depended jointly on their body size and metabolic profile [44, 45]. Furthermore, our study indicated that HTGW was a stable risk factor for diabetes in all stratified analyses, implicating that hypertriglyceridemic-waist phenotype might be a good and stable indicator of visceral adiposity in hypertensive patients.

The TyG index, derived from TG and FPG, has been used as a marker to identify insulin resistance in many epidemiological studies. A study conducted in the Mexican population showed that the TyG index performed high sensitivity (96.5%) and specificity (85.0%), compared with the hyperinsulinemic-euglycemic clamp test [16]; another study conducted in the Brazilian population indicated that the TyG index was better than homeostasis model assessment in identifying patients with insulin resistance [46]; a real-world study suggested that compared with homeostasis model assessment for insulin resistance, the TyG index is more strongly associated with arterial stiffness in T2DM patients [47]. A recent meta-analysis included 14 studies showed that TyG index was associated with an increased risk of T2DM in a nonlinear fashion, which is consistent with our study [24]. Li et al. also found that TyG index was positively associated with the risk of diabetes in the subjects with SBP ≥ 140 mmHg and DBP ≥ 90 mmHg after conducting subgroup analyses among Chinese [48]. In our study, we found that the positive relationship between TyG index and T2DM risk was consistent in all subgroups among hypertension patients, and seemed to be more evident in younger subjects or in non-retirees. This result was reasonable because the retirees tended to be older and with poorer physical functioning [49]. Based on the stratified analysis, our findings suggested that among hypertension patients, the TyG index may be promising for screening risk of T2DM, especially in subjects without high-risk factors, such as retirement and older age, which expanded the results of previous study conducted in apparently healthy adults [48].

The LAP was reported as a reliable visceral adiposity index combining WC and TG levels [18]. Several studies have found that LAP was a better predictor of metabolic syndrome and diabetes than the common obesity index [50–52]. A recent cohort study conducted among 15,717 Chinese participants showed that high LAP was associated with high risk of developing T2DM, suggesting that LAP may be a potential indicator for T2DM [26]. Similar results were also found in Japanese and Korean populations [53, 54]. Our study indicated that the positive relationship between LAP and T2DM risk was in a nonlinear fashion among hypertension patients, which is also consistent with prior evidence conducted in the general populations [26]. The VAI is another reliable lipid index to estimate insulin resistance from visceral adiposity dysfunction [19]. A meta-analysis included ten observational studies involving 112,603 participants found that VAI may be related to a high risk of prediabetes [55]. A recent meta-analysis included nine cohort studies showed that each 1-unit increment of VAI was associated with 42% higher risk of T2DM [25]. Yu et al. also conducted a cohort analysis and found that high-VAI level was associated with increased risk of new-onset T2DM [56]. Expanded the notions of previous studies, our study indicated that increased LAP and VAI levels were associated with increased risk of T2DM among subjects with hypertension, and these positive relationships persisted in all stratification variables, which suggested that LAP and VAI may serve as early markers of T2DM risk in hypertensive patients.

Although the exact mechanisms for the relationship of these indicators with T2DM among hypertensive patients are unclear, several hypotheses were proposed. Increased visceral adipose is linked to an increased risk of insulin resistance and β-cell dysfunction [55]. As the markers of visceral adiposity, increased LAP, VAI, and enlarged WC could promote the accumulation of various inflammatory cytokines, which could accelerate the progression of insulin resistance and glucose intolerance and cause the development of diabetes directly [57]. Additionally, excessive adipose tissue could produce excessive free fatty acids, which may deteriorate insulin sensitivity and exacerbate T2DM risk subsequently [58]. Moreover, the increased TG level in the blood could lead to the inhibited insulin activity and muscle catabolism, and overloaded TG in the pancreatic islet cells could also lead to β-cell dysfunction [59]. And elevated glucose concentrations could raise the level of reactive oxygen species and exert toxic effects on β-cells [60]. High TyG index, as the product of TG and FPG, reflected decreased β-cells and the increased IR, which contributed to the development of T2DM [61]. As a common comorbidity of diabetes, hypertension is a major risk factor for T2DM [62]. Our analyses demonstrated that HTGW, TyG, LAP, and VAI were positively associated with T2DM, even when the subjects recruited were limited to those with high risk of T2DM, which further confirmed the possibility that these indicators may serve as novel and simple non-invasive markers to evaluate the risk of T2DM. More mechanistic researches are needed to reveal the role of these indicators in the development of diabetes among hypertensive patients.

Our study has several strengths. First, the present study examined the associations of four novel indicators with the risk of T2DM among a large number of hypertensive participants, and thus, the results are specifically applicable and dependable for the participants with hypertension. Second, we have taken TyG, LAP, and VAI as both continuous variable and categorical variable when doing the analysis, and sensitivity analysis and trend examination were performed to improve the reliability of the results. Finally, we conducted subgroup analyses and interaction test to enhance the credibility of the results and identify potential interactions with other variables.

There are also some limitations. First, this is a cross-sectional study, which limited the ability of determining the direction of the association and inferring a causal association. Second, the diagnosis of T2DM was determined by self-reported or fasting blood glucose. A lack of oral glucose tolerance test and determination of glycosylated haemoglobin might underestimate the strength of association. Third, all the participants included in the present study were Chinese adults with hypertension, which made it more useful for specific patients.

In conclusion, the results found that among hypertensive patients, HTGW and higher level of TyG, LAP, and VAI were associated with the elevated risk of T2DM. The findings suggest that HTGW, TyG, LAP, and VAI may serve as simple and effective tools for T2DM risk assessment in the prevention and management of main chronic diseases.

## Supplementary Information

**Additional file 1:** **Figure S1.** Flow chart of selection of hypertensive patients. **Table S1.** Association between lipid indicators and the risk of T2DM. **Table S2.** Stratified analyses on the association between triglyceridemic-waist phenotypes and the risk of T2DM. **Table S3.** Stratified analyses on the association between triglyceride glucose index and the risk of T2DM. **Table S4.** Stratified analyses on the association between lipid accumulation product and the risk of T2DM. **Table S5.** Stratified analyses on the association between visceral adiposity index and the risk of T2DM. **Table S6. **Association between triglyceridemic-waist phenotypes and the risk of T2DM by defining WC ≥ 80 cm in women as enlarged waist circumference. **Table S7. **Association between lipid indices and the risk of T2DM with additional adjustment for total cholesterol and LDL-C. **Table S8. **Association between lipid indicators and the risk of T2DM by using different definition of hypertension.

## Data Availability

The data used to support the findings of this study are available from the corresponding author upon request. A proposal with detailed description of study objectives and statistical analysis plan will be needed for evaluation of the reasonability of requests if someone requests data sharing.

## Abbreviations

BMI: Body mass index
CI: Confidence interval
DBP: Diastolic blood pressure
FPG: Fasting plasma glucose
HDL-C: High-density lipoprotein cholesterol
HTGW: Elevated triglyceride level and enlarged waist circumference
HTNW: Elevated triglyceride level and normal waist circumference
IQR: Interquartile range
LAP: Lipid accumulation product
LDL-C: Low-density lipoprotein cholesterol
NTNW: Normal triglyceride level and normal waist circumference
NTGW: Normal triglyceride level and enlarged waist circumference
OR: Odds ratios
SBP: Systolic blood pressure
SD: Standard deviation
T2DM: Type 2 diabetes mellitus
TG: Triglyceride
TyG index: Triglyceride glucose index
VAI: Visceral adiposity index
WC: Waist circumference
