# Correction: Novel lipid indicators and the risk of type 2 diabetes mellitus among Chinese hypertensive patients: findings from the Guangzhou Heart Study

**DOI:** 10.1186/s12933-022-01731-1

**Published:** 2023-01-11

**Authors:** Hai Deng, Peng Hu, Huoxing Li, Huanning Zhou, Xiuyi Wu, Maohua Yuan, Xueru Duan, Miaochan Lao, Chuchu Wu, Murui Zheng, Xiang Qian Lao, Wenjing Zhao, Xudong Liu

**Affiliations:** 1 Department of Cardiology, Guangdong Cardiovascular Institute, Guangdong Provincial People’s Hospital, Guangdong Academy of Medical Science, Guangzhou, 510080 China; 2 School of Public Health, Guangdong Pharmaceutical University, No. 283 Jianghai Avenue, Haizhu District, Guangzhou, 510310 China; 3 Department of Epidemiology, School of Public Health, Sun Yat-Sen University, Guangzhou, 510080 China; 4 The Second School of Clinical Medicine, Southern Medical University, Guangzhou, 510515 China; 5 Guangzhou Yuexiu District Center for Disease Control and Prevention, Guangzhou, China; 6 Nancun Community Health Service Center, Guangzhou, 511442 China; 7 Dadong Street Community Health Service Center, Guangzhou, 510080 China; 8 Department of Sleep Center, Department of Geriatric Respiratory, Guangdong Provincial People’s Hospital, Guangdong Academy of Medical Sciences, Guangdong Provincial Geriatrics Institute, Guangzhou, 510080 China; 9 Department of Community Health, Guangzhou Center for Disease Control and Prevention, No. 1 Qide Road, Baiyun District, Guangzhou, 510440 China; 10 JC School of Public Health and Primary Care, Faculty of Medicine, The Chinese University of Hong Kong, Hong Kong, 999077 SAR China; 11 School of Public Health and Emergency Management, Southern University of Science and Technology, No. 1088 Xueyuan Avenue, Shenzhen, 518055 China

**Correction: Cardiovascular Diabetology (2022) 21:212** **https://doi.org/10.1186/s12933-022-01660-z**

Following publication of the original article [1], the author noticed an error in the given name of the co-author. The eighth author’s name should be “Miaochan Lao” rather than “Miaochao Lao”. This has been corrected with this erratum.

The original article has also been corrected.
