# Supplementary material for: Novel lipid indicators and the risk of type 2 diabetes mellitus among Chinese hypertensive patients: findings from the Guangzhou Heart Study
Source: Cardiovasc Diabetol. 2022 Oct 15;21:212. doi: 10.1186/s12933-022-01660-z (PMC9571423; doi:10.1186/s12933-022-01660-z)
Supplement: Supplementary file 1 — Additional file 1: Figure S1. Flow chart of selection of hypertensive patients. Table S1. Association between lipid indicators and the risk of T2DM. Table S2. Stratified analyses on the association between triglyceridemic-waist phenotypes and the risk of T2DM. Table S3. Stratified analyses on the association between triglyceride glucose index and the risk of T2DM. Table S4. Stratified analyses on the association between lipid accumulation product and the risk of T2DM. Table S5. Stratified analyses on the association between visceral adiposity index and the risk of T2DM. Table S6. Association between triglyceridemic-waist phenotypes and the risk of T2DM by defining WC ≥ 80 cm in women as enlarged waist circumference. Table S7. Association between lipid indices and the risk of T2DM with additional adjustment for total cholesterol and LDL-C. Table S8. Association between lipid indicators and the risk of T2DM by using different definition of hypertension. [file 12933_2022_1660_MOESM1_ESM.docx]

**Additional file 1**

**Novel lipid indicators and the risk of type 2 diabetes among Chinese hypertensive patients: findings from the Guangzhou Heart Study**

**Supplementary Figure S1.** Flow chart of selection of hypertensive patients

**Supplementary Table S1.** Association between lipid indicators and the risk of T2DM

**Supplementary Table S2.** Stratified analyses on the association between triglyceridemic-waist phenotypes and the risk of T2DM

**Supplementary Table S3.** Stratified analyses on the association between triglyceride glucose index and the risk of T2DM

**Supplementary Table S4.** Stratified analyses on the association between lipid accumulation product and the risk of T2DM

**Supplementary Table S5.** Stratified analyses on the association between visceral adiposity index and the risk of T2DM

**Supplementary Table S6.** Association between triglyceridemic-waist phenotypes and the risk of T2DM by defining WC ≥ 80 cm in women as enlarged waist circumference

**Supplementary Table S7.** Association between lipid indices and the risk of T2DM with additional adjustment for total cholesterol and LDL-C

**Supplementary Table S8.** Association between lipid indicators and the risk of T2DM by using different definition of hypertension


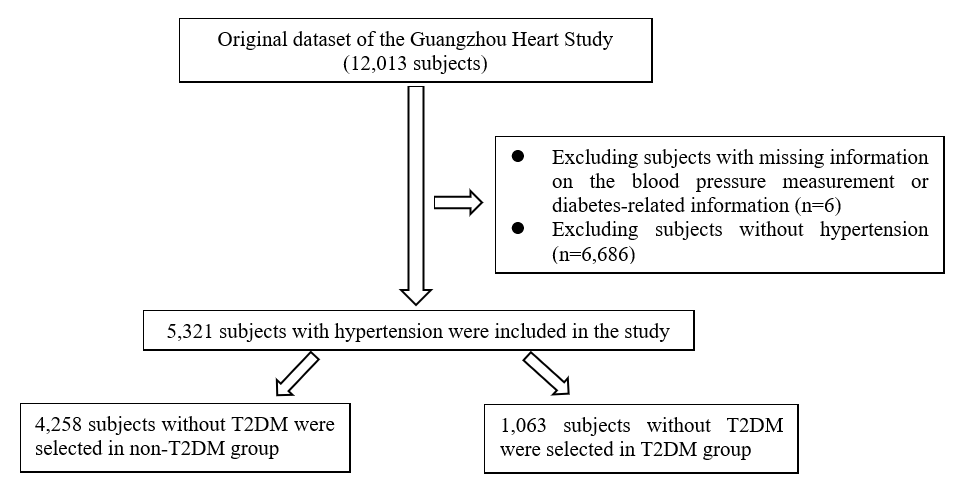


Figure S1. Flow chart of selection of hypertensive patients

Table S1. Association between lipid indicators and the risk of T2DM

|  | N^*^ | |  | OR (95% CI) | |
| --- | --- | --- | --- | --- | --- |
|  | Non-T2DM group | T2DM group |  | Crude | Adjusted^†^ |
| Hypertriglyceridemic-waist phenotype | | | | | |
| NTNW | 1412 | 216 |  | 1.00 | 1.00 |
| NTGW | 1309 | 343 |  | 1.71 (1.42, 2.07) | 1.62 (1.30, 2.02) |
| HTNW | 622 | 152 |  | 1.60 (1.27, 2.00) | 1.73 (1.37, 2.19) |
| HTGW | 915 | 352 |  | 2.51 (2.08, 3.04) | 2.57 (2.05, 3.23) |
| TyG |  |  |  |  |  |
| Quartile 1 | 1185 | 145 |  | 1.00 | 1.00 |
| Quartile 2 | 1153 | 177 |  | 1.25 (0.99, 1.59) | 1.26 (0.99, 1.60) |
| Quartile 3 | 1088 | 243 |  | 1.83 (1.46, 2.28) | 1.88 (1.50, 2.37) |
| Quartile 4 | 832 | 498 |  | 4.89 (3.99, 6.02) | 5.35 (4.33, 6.64) |
| *P* for trend |  |  |  | <0.001 | <0.001 |
| Every 1-unit increment |  |  |  | 1.76 (1.65, 1.88) | 1.81 (1.69, 1.94) |
| LAP |  |  |  |  |  |
| Quartile 1 | 1151 | 179 |  | 1.00 | 1.00 |
| Quartile 2 | 1097 | 233 |  | 1.37 (1.11, 1.69) | 1.39 (1.11, 1.73) |
| Quartile 3 | 1050 | 280 |  | 1.71 (1.40, 2.11) | 1.76 (1.41, 2.21) |
| Quartile 4 | 960 | 371 |  | 2.48 (2.04, 3.03) | 2.65 (2.11, 3.34) |
| *P* for trend |  |  |  | <0.001 | <0.001 |
| Every 1-unit increment |  |  |  | 1.35 (1.27, 1.43) | 1.38 (1.28, 1.48) |
| VAI |  |  |  |  |  |
| Quartile 1 | 1124 | 206 |  | 1.00 | 1.00 |
| Quartile 2 | 1113 | 217 |  | 1.06 (0.86, 1.31) | 1.11 (0.90, 1.38) |
| Quartile 3 | 1052 | 279 |  | 1.45 (1.19, 1.77) | 1.50 (1.21, 1.84) |
| Quartile 4 | 969 | 361 |  | 2.03 (1.68, 2.46) | 2.17 (1.77, 2.67) |
| *P* for trend |  |  |  | <0.001 | <0.001 |
| Every 1-unit increment |  |  |  | 1.29 (1.21, 1.37) | 1.31 (1.23, 1.40) |

Abbreviations: HTGW, elevated triglyceride level and enlarged waist circumference; HTNW, elevated triglyceride level and normal waist circumference; NTGW, normal triglyceride level and enlarged waist circumference; NTNW, normal triglyceride level and normal waist circumference; TyG, triglyceride-glucose; LAP, lipid accumulation product; VAI, visceral adiposity index.

^*^ N represents sample size for non- diabetes group or for diabetes group.

^†^ Adjustment for age, sex, education, marital status, retirement status, smoking, alcohol drinking, leisure-time physical activity, body mass index, systolic blood pressure, diastolic blood pressure, and history of cardiovascular diseases.

Table S2. Stratified analyses on the association between triglyceridemic-waist phenotypes and the risk of T2DM

|  | N^*^ | |  | OR (95% CI) ^†^ | | | |  | *P*-_interaction_ |
| --- | --- | --- | --- | --- | --- | --- | --- | --- | --- |
|  | Non-diabetes | Diabetes |  | NTNW | NTGW | HTNW | HTGW |  |  |
| Age group |  |  |  |  |  |  |  |  | 0.314 |
| < 60 years | 1443 | 261 |  | 1.00 | 1.44 (0.90, 2.29) | 2.04 (1.31, 3.18) | 2.68 (1.71, 4.25) |  |  |
| ≥ 60 years | 2815 | 802 |  | 1.00 | 1.70 (1.33, 2.19) | 1.61 (1.22, 2.12) | 2.49 (1.91, 3.24) |  |  |
| Sex |  |  |  |  |  |  |  |  | 0.805 |
| Male | 1665 | 413 |  | 1.00 | 1.77 (1.24, 2.53) | 1.94 (1.36, 2.76) | 2.91 (2.02, 4.21) |  |  |
| Female | 2593 | 650 |  | 1.00 | 1.49 (1.12, 1.98) | 1.61 (1.18, 2.19) | 2.28 (1.70, 3.06) |  |  |
| Retirement status |  |  |  |  |  |  |  |  | 0.443 |
| Retirement | 2919 | 847 |  | 1.00 | 1.69 (1.32, 2.16) | 1.73 (1.33, 2.24) | 2.47 (1.91, 3.20) |  |  |
| Non-retirement | 1339 | 216 |  | 1.00 | 1.26 (0.77, 2.09) | 1.78 (1.06, 2.96) | 2.64 (1.63, 4.33) |  |  |
| Education |  |  |  |  |  |  |  |  | 0.630 |
| < High school | 2944 | 724 |  | 1.00 | 1.64 (1.28, 2.16) | 1.76 (1.34, 2.36) | 2.59 (1.97, 3.42) |  |  |
| ≥ High school | 1314 | 339 |  | 1.00 | 1.63 (1.13, 2.38) | 1.69 (1.14, 2.49) | 2.50 (1.67, 3.76) |  |  |
| BMI |  |  |  |  |  |  |  |  | 0.008 |
| < 24 kg/m^2^ | 1815 | 380 |  | 1.00 | 1.71 (1.21, 2.38) | 2.15 (1.64, 2.82) | 2.21 (1.45, 3.31) |  |  |
| ≥ 24 kg/m^2^ | 2443 | 683 |  | 1.00 | 1.58 (1.15, 2.20) | 1.04 (0.65, 1.66) | 2.57 (1.87, 3.59) |  |  |
| Smoking |  |  |  |  |  |  |  |  | 0.153 |
| No | 3272 | 835 |  | 1.00 | 1.44 (1.13, 1.84) | 1.54 (1.18, 2.01) | 2.36 (1.83, 3.06) |  |  |
| Yes | 986 | 228 |  | 1.00 | 2.45 (1.50, 4.05) | 2.57 (1.57, 4.22) | 3.36 (2.05, 5.60) |  |  |
| Alcohol drinking |  |  |  |  |  |  |  |  | 0.137 |
| No | 3375 | 877 |  | 1.00 | 1.49 (1.17, 1.90) | 1.80 (1.39, 2.33) | 2.28 (1.77, 2.94) |  |  |
| Yes | 883 | 186 |  | 1.00 | 2.33 (1.37, 4.00) | 1.52 (0.85, 2.69) | 4.11 (2.42, 7.09) |  |  |

Abbreviations: BMI, body mass index; HTGW, elevated triglyceride level and enlarged waist circumference; HTNW, elevated triglyceride level and normal waist circumference; NTGW, normal triglyceride level and enlarged waist circumference; NTNW, normal triglyceride level and normal waist circumference.

^*^ N represents sample size for non- diabetes group or for diabetes group.

^†^ Adjustment for age, sex, education, marital status, retirement status, smoking, alcohol drinking, leisure-time physical activity, body mass index, systolic blood pressure, diastolic blood pressure, and history of cardiovascular diseases except the corresponding stratification variable.

Table S3. Stratified analyses on the association between triglyceride glucose index and the risk of T2DM

|  | N^*^ | |  | OR (95% CI) ^†^ | | | |  | *P* for trend | *P*-_interaction_ |
| --- | --- | --- | --- | --- | --- | --- | --- | --- | --- | --- |
|  | Non-diabetes | Diabetes |  | Quartile 1 | Quartile 2 | Quartile 3 | Quartile 4 |  |  |  |
| Age group |  |  |  |  |  |  |  |  |  | 0.006 |
| < 60 years | 1443 | 261 |  | 1.00 | 1.19 (0.66, 2.16) | 2.71 (1.64, 4.62) | 8.71 (5.45, 14.52) |  | <0.001 |  |
| ≥ 60 years | 2815 | 802 |  | 1.00 | 1.27 (0.98, 1.65) | 1.70 (1.31, 2.20) | 4.48 (3.53, 5.71) |  | <0.001 |  |
| Sex |  |  |  |  |  |  |  |  |  | 0.181 |
| Male | 1665 | 413 |  | 1.00 | 1.45 (0.98, 2.14) | 2.54 (1.76, 3.70) | 6.48 (4.58, 9.29) |  | <0.001 |  |
| Female | 2593 | 650 |  | 1.00 | 1.13 (0.83, 1.53) | 1.53 (1.15, 2.05) | 4.65 (3.56, 6.12) |  | <0.001 |  |
| Retirement status |  |  |  |  |  |  |  |  |  | 0.001 |
| Retirement | 2919 | 847 |  | 1.00 | 1.27 (0.98, 1.64) | 1.61 (1.25, 2.07) | 4.66 (3.70, 5.91) |  | <0.001 |  |
| Non-retirement | 1339 | 216 |  | 1.00 | 1.37 (0.69, 2.82) | 4.04 (2.25, 7.73) | 10.65 (6.08, 20.06) |  | <0.001 |  |
| Education |  |  |  |  |  |  |  |  |  | 0.434 |
| < High school | 2944 | 724 |  | 1.00 | 1.41 (1.04, 1.91) | 1.99 (1.49, 2.67) | 5.95 (4.55, 7.85) |  | <0.001 |  |
| ≥ High school | 1314 | 339 |  | 1.00 | 1.01 (0.68, 1.49) | 1.71 (1.18, 2.49) | 4.22 (2.98, 6.04) |  | <0.001 |  |
| BMI |  |  |  |  |  |  |  |  |  | 0.442 |
| < 24 kg/m^2^ | 1815 | 380 |  | 1.00 | 1.03 (0.73, 1.47) | 1.82 (1.29, 2.56) | 5.22 (3.80, 7.24) |  | <0.001 |  |
| ≥ 24 kg/m^2^ | 2443 | 683 |  | 1.00 | 1.49 (1.08, 2.08) | 2.04 (1.51, 2.80) | 5.78 (4.34, 7.77) |  | <0.001 |  |
| Smoking |  |  |  |  |  |  |  |  |  | 0.523 |
| No | 3272 | 835 |  | 1.00 | 1.19 (0.91, 1.56) | 1.84 (1.43, 2.38) | 4.99 (3.94, 6.36) |  | <0.001 |  |
| Yes | 986 | 228 |  | 1.00 | 1.48 (0.88, 2.53) | 2.04 (1.21, 3.48) | 6.51 (4.10, 10.68) |  | <0.001 |  |
| Alcohol drinking |  |  |  |  |  |  |  |  |  | 0.352 |
| No | 3375 | 877 |  | 1.00 | 1.22 (0.94, 1.59) | 1.74 (1.36, 2.24) | 5.30 (4.20, 6.73) |  | <0.001 |  |
| Yes | 883 | 186 |  | 1.00 | 1.44 (0.79, 2.64) | 2.67 (1.55, 4.73) | 5.69 (3.46, 9.73) |  | <0.001 |  |

^*^ N represents sample size for non- diabetes group or for diabetes group.

^†^ Adjustment for age, sex, education, marital status, retirement status, smoking, alcohol drinking, leisure-time physical activity, body mass index, systolic blood pressure, diastolic blood pressure, and history of cardiovascular diseases except the corresponding stratification variable.

Table S4. Stratified analyses on the association between lipid accumulation product and the risk of T2DM

|  | N^*^ | |  | OR (95% CI) ^†^ | | | |  | *P* for trend | *P*-_interaction_ |
| --- | --- | --- | --- | --- | --- | --- | --- | --- | --- | --- |
|  | Non-diabetes | Diabetes |  | Quartile 1 | Quartile 2 | Quartile 3 | Quartile 4 |  |  |  |
| Age group |  |  |  |  |  |  |  |  |  | 0.655 |
| < 60 years | 1443 | 261 |  | 1.00 | 1.51 (0.94, 2.47) | 2.19 (1.38, 3.53) | 3.42 (2.14, 5.56) |  | <0.001 |  |
| ≥ 60 years | 2815 | 802 |  | 1.00 | 1.33 (1.03, 1.71) | 1.63 (1.26, 2.12) | 2.35 (1.80, 3.07) |  | <0.001 |  |
| Sex |  |  |  |  |  |  |  |  |  | 0.802 |
| Male | 1665 | 413 |  | 1.00 | 1.51 (1.08, 2.11) | 2.05 (1.46, 2.90) | 2.95 (2.06, 4.24) |  | <0.001 |  |
| Female | 2593 | 650 |  | 1.00 | 1.25 (0.93, 1.69) | 1.53 (1.13, 2.07) | 2.31 (1.70, 3.15) |  | <0.001 |  |
| Retirement status |  |  |  |  |  |  |  |  |  | 0.509 |
| Retirement | 2919 | 847 |  | 1.00 | 1.39 (1.09, 1.78) | 1.77 (1.38, 2.28) | 2.48 (1.91, 3.23) |  | <0.001 |  |
| Non-retirement | 1339 | 216 |  | 1.00 | 1.27 (0.76, 2.14) | 1.64 (0.98, 2.76) | 3.03 (1.84, 5.07) |  | <0.001 |  |
| Education |  |  |  |  |  |  |  |  |  | 0.498 |
| < High school | 2944 | 724 |  | 1.00 | 1.50 (1.13, 1.99) | 1.77 (1.33, 2.35) | 2.68 (2.01, 3.59) |  | <0.001 |  |
| ≥ High school | 1314 | 339 |  | 1.00 | 1.23 (0.85, 1.77) | 1.81 (1.25, 2.65) | 2.52 (1.70, 3.75) |  | <0.001 |  |
| BMI |  |  |  |  |  |  |  |  |  | 0.426 |
| < 24 kg/m^2^ | 1815 | 380 |  | 1.00 | 1.31 (0.98, 1.75) | 2.01 (1.47, 2.74) | 2.44 (1.69, 3.48) |  | <0.001 |  |
| ≥ 24 kg/m^2^ | 2443 | 683 |  | 1.00 | 1.53 (1.07, 2.24) | 1.79 (1.26, 2.58) | 2.91 (2.07, 4.17) |  | <0.001 |  |
| Smoking |  |  |  |  |  |  |  |  |  | 0.400 |
| No | 3272 | 835 |  | 1.00 | 1.30 (1.01, 1.67) | 1.59 (1.23, 2.06) | 2.53 (1.95, 3.30) |  | <0.001 |  |
| Yes | 986 | 228 |  | 1.00 | 1.66 (1.04, 2.67) | 2.37 (1.49, 3.82) | 2.87 (1.77, 4.70) |  | <0.001 |  |
| Alcohol drinking |  |  |  |  |  |  |  |  |  | 0.369 |
| No | 3375 | 877 |  | 1.00 | 1.27 (0.99, 1.62) | 1.64 (1.28, 2.10) | 2.40 (1.86, 3.12) |  | <0.001 |  |
| Yes | 883 | 186 |  | 1.00 | 1.94 (1.14, 3.34) | 2.31 (1.36, 4.01) | 3.81 (2.25, 6.56) |  | <0.001 |  |

^*^ N represents sample size for non- diabetes group or for diabetes group.

^†^ Adjustment for age, sex, education, marital status, retirement status, smoking, alcohol drinking, leisure-time physical activity, body mass index, systolic blood pressure, diastolic blood pressure, and history of cardiovascular diseases except the corresponding stratification variable.

Table S5. Stratified analyses on the association between visceral adiposity index and the risk of T2DM

|  | N^*^ | |  | OR (95% CI) ^†^ | | | |  | *P* for trend | *P*-_interaction_ |
| --- | --- | --- | --- | --- | --- | --- | --- | --- | --- | --- |
|  | Non-diabetes | Diabetes |  | Quartile 1 | Quartile 2 | Quartile 3 | Quartile 4 |  |  |  |
| Age group |  |  |  |  |  |  |  |  |  | 0.741 |
| < 60 years | 1443 | 261 |  | 1.00 | 1.00 (0.63, 1.57) | 1.63 (1.08, 2.50) | 2.21 (1.47, 3.37) |  | <0.001 |  |
| ≥ 60 years | 2815 | 802 |  | 1.00 | 1.13 (0.88, 1.44) | 1.38 (1.08, 1.76) | 2.07 (1.63, 2.62) |  | <0.001 |  |
| Sex |  |  |  |  |  |  |  |  |  | 0.258 |
| Male | 1665 | 413 |  | 1.00 | 1.13 (0.83, 1.54) | 1.78 (1.30, 2.43) | 2.55 (1.86, 3.50) |  | <0.001 |  |
| Female | 2593 | 650 |  | 1.00 | 1.03 (0.76, 1.39) | 1.25 (0.94, 1.67) | 1.83 (1.39, 2.42) |  | <0.001 |  |
| Retirement status |  |  |  |  |  |  |  |  |  | 0.874 |
| Retirement | 2919 | 847 |  | 1.00 | 1.08 (0.85, 1.38) | 1.38 (1.10, 1.75) | 2.08 (1.65, 2.62) |  | <0.001 |  |
| Non-retirement | 1339 | 216 |  | 1.00 | 1.24 (0.76, 2.02) | 1.85 (1.17, 2.98) | 2.51 (1.59, 4.01) |  | <0.001 |  |
| Education |  |  |  |  |  |  |  |  |  | 0.814 |
| < High school | 2944 | 724 |  | 1.00 | 1.11 (0.85, 1.46) | 1.52 (1.17, 1.98) | 2.09 (1.62, 2.71) |  | <0.001 |  |
| ≥ High school | 1314 | 339 |  | 1.00 | 1.09 (0.77, 1.55) | 1.41 (1.00, 2.00) | 2.24 (1.58, 3.19) |  | <0.001 |  |
| Body mass index |  |  |  |  |  |  |  |  |  | 0.625 |
| < 24 kg/m^2^ | 1815 | 380 |  | 1.00 | 0.99 (0.71, 1.37) | 1.59 (1.15, 2.18) | 2.34 (1.69, 3.25) |  | <0.001 |  |
| ≥ 24 kg/m^2^ | 2443 | 683 |  | 1.00 | 1.22 (0.92, 1.63) | 1.53 (1.16, 2.03) | 2.19 (1.68, 2.87) |  | <0.001 |  |
| Smoking |  |  |  |  |  |  |  |  |  | 0.644 |
| No | 3272 | 835 |  | 1.00 | 1.07 (0.83, 1.37) | 1.38 (1.09, 1.76) | 2.03 (1.60, 2.57) |  | <0.001 |  |
| Yes | 986 | 228 |  | 1.00 | 1.17 (0.76, 1.8) | 1.77 (1.15, 2.72) | 2.53 (1.66, 3.87) |  | <0.001 |  |
| Alcohol drinking |  |  |  |  |  |  |  |  |  | 0.181 |
| No | 3375 | 877 |  | 1.00 | 1.08 (0.85, 1.38) | 1.36 (1.08, 1.72) | 2.15 (1.71, 2.71) |  | <0.001 |  |
| Yes | 883 | 186 |  | 1.00 | 1.21 (0.74, 1.96) | 2.20 (1.38, 3.52) | 2.19 (1.38, 3.49) |  | <0.001 |  |

^*^ N represents sample size for non- diabetes group or for diabetes group.

^†^ Adjustment for age, sex, education, marital status, retirement status, smoking, alcohol drinking, leisure-time physical activity, body mass index, systolic blood pressure, diastolic blood pressure, and history of cardiovascular diseases except the corresponding stratification variable.

Table S6 Association between triglyceridemic-waist phenotypes and the risk of T2DM by defining WC ≥ 80 cm in women as enlarged waist circumference

|  | **N^*^** | |  | **OR (95% CI)** | |
| --- | --- | --- | --- | --- | --- |
|  | **Non-diabetes group** | **Diabetes group** |  | **Crude** | **Adjusted** ^†^ |
| NTNW | 1052 | 151 |  | 1.00 | 1.00 |
| NTGW | 1669 | 408 |  | 1.70 (1.39, 2.09) | 1.59 (1.26, 2.01) |
| HTNW | 413 | 111 |  | 1.87 (1.43, 2.45) | 2.03 (1.53, 2.67) |
| HTGW | 1124 | 393 |  | 2.44 (1.99, 3.00) | 2.46 (1.91, 3.10) |

Abbreviations: HTGW, elevated triglyceride level and enlarged waist circumference; HTNW, elevated triglyceride level and normal waist circumference; NTGW, normal triglyceride level and enlarged waist circumference; NTNW, normal triglyceride level and normal waist circumference; WC, waist circumference.

^*^ N represents sample size for non- diabetes group or for diabetes group.

^†^ Adjustment for age, sex, education, marital status, retirement status, smoking, alcohol drinking, leisure-time physical activity, body mass index, systolic blood pressure, diastolic blood pressure, and history of cardiovascular diseases.

Table S7 Association between lipid indices and the risk of T2DM with additional adjustment for total cholesterol and LDL-C

|  | **Model 1^*^** | **Model 2**^†^ | **Model 3** ^‡^ | **Model 4** ^§^ |
| --- | --- | --- | --- | --- |
|  | **OR (95% CI)** | **OR (95% CI)** | **OR (95% CI)** | **OR (95% CI)** |
| Triglyceridemic-waist phenotype |  |  |  |  |
| NTNW | 1.00 | 1.00 | 1.00 | 1.00 |
| NTGW | 1.62 (1.30, 2.02) | 1.62 (1.30, 2.02) | 1.62 (1.30, 2.02) | 1.63 (1.31, 2.03) |
| HTNW | 1.73 (1.37, 2.19) | 1.71 (1.35, 2.16) | 1.74 (1.38, 2.20) | 1.67 (1.32, 2.11) |
| HTGW | 2.57 (2.05, 3.23) | 2.54 (2.02, 3.19) | 2.59 (2.06, 3.25) | 2.49 (1.98, 3.14) |
| TyG |  |  |  |  |
| Q1 | 1.00 | 1.00 | 1.00 | 1.00 |
| Q2 | 1.26 (0.99, 1.60) | 1.28 (1.01, 1.62) | 1.29 (1.01, 1.63) | 1.28 (1.01, 1.63) |
| Q3 | 1.88 (1.50, 2.37) | 1.92 (1.53, 2.42) | 1.94 (1.54, 2.45) | 1.94 (1.54, 2.44) |
| Q4 | 5.35 (4.33, 6.64) | 5.53 (4.46, 6.90) | 5.49 (4.43, 6.82) | 5.51 (4.44, 6.88) |
| *P* for trend | <0.001 | <0.001 | <0.001 | <0.001 |
| Every 1-unit increment | 1.81 (1.69, 1.94) | 1.83 (1.71, 1.97) | 1.83 (1.71, 1.96) | 1.82 (1.70, 1.95) |
| LAP |  |  |  |  |
| Q1 | 1.00 | 1.00 | 1.00 | 1.00 |
| Q2 | 1.39 (1.11, 1.73) | 1.38 (1.11, 1.73) | 1.39 (1.12, 1.74) | 1.40 (1.12, 1.75) |
| Q3 | 1.76 (1.41, 2.21) | 1.75 (1.39, 2.19) | 1.78 (1.42, 2.24) | 1.78 (1.42, 2.24) |
| Q4 | 2.65 (2.11, 3.34) | 2.61 (2.07, 3.31) | 2.67 (2.12, 3.38) | 2.53 (2.00, 3.21) |
| *P* for trend | <0.001 | <0.001 | <0.001 | <0.001 |
| Every 1-unit increment | 1.38 (1.28, 1.48) | 1.37 (1.27, 1.48) | 1.38 (1.28, 1.49) | 1.35 (1.26, 1.46) |
| VAI |  |  |  |  |
| Q1 | 1.00 | 1.00 | 1.00 | 1.00 |
| Q2 | 1.11 (0.90, 1.38) | 1.11 (0.89, 1.37) | 1.11 (0.90, 1.38) | 1.17 (0.94, 1.45) |
| Q3 | 1.50 (1.21, 1.84) | 1.48 (1.20, 1.83) | 1.50 (1.21, 1.85) | 1.59 (1.28, 1.97) |
| Q4 | 2.17 (1.77, 2.67) | 2.14 (1.74, 2.63) | 2.17 (1.77, 2.67) | 2.14 (1.75, 2.64) |
| *P* for trend | <0.001 | <0.001 | <0.001 | <0.001 |
| Every 1-unit increment | 1.31 (1.23, 1.40) | 1.30 (1.22, 1.39) | 1.31 (1.23, 1.40) | 1.30 (1.22, 1.39) |

Abbreviations: HTGW, elevated triglyceride level and enlarged waist circumference; HTNW, elevated triglyceride level and normal waist circumference; NTGW, normal triglyceride level and enlarged waist circumference; NTNW, normal triglyceride level and normal waist circumference; LAP, lipid accumulation product; TyG, triglyceride-glucose; VAI, visceral adiposity index; LDL-C, low density lipoprotein cholesterol.

^*^ In model 1, OR was adjusted for age, sex, education, marital status, retirement status, smoking, alcohol drinking, leisure-time physical activity, body mass index, systolic blood pressure, diastolic blood pressure, and history of cardiovascular diseases.

^†^ In Model 2, OR was adjusted for covariates in model and total cholesterol.

^‡^ In Model 3, OR was adjusted for covariates in model and LDL-C.

^§^ In Model 4, OR was adjusted for covariates in model, total cholesterol and LDL-C.

Table S8 Association between lipid indicators and the risk of T2DM by using different definition of hypertension (N=6784)

|  | **N^*^** | |  | **OR (95% CI)** | |
| --- | --- | --- | --- | --- | --- |
|  | **Non-T2DM group** | **T2DM group** |  | **Crude** | **Adjusted^†^** |
| Hypertriglyceridemic-waist phenotype | | | | | |
| NTNW | 1958 | 262 |  | 1.00 | 1.00 |
| NTGW | 1649 | 391 |  | 1.77 (0.50, 2.10) | 1.53 (1.25, 1.87) |
| HTNW | 821 | 193 |  | 1.76 (1.43, 2.15) | 1.83 (1.49, 2.26) |
| HTGW | 1117 | 393 |  | 3.63 (2.21, 3.13) | 2.40 (1.94, 2.96) |
| TyG |  |  |  |  |  |
| Quartile 1 | 1537 | 159 |  | 1.00 | 1.00 |
| Quartile 2 | 1502 | 194 |  | 1.25 (1.00, 1.56) | 1.26 (1.01, 1.58) |
| Quartile 3 | 1414 | 282 |  | 1.93 (1.57, 2.38) | 1.97 (1.59, 2.44) |
| Quartile 4 | 1092 | 604 |  | 5.35 (4.43, 6.49) | 5.87 (4.80, 7.21) |
| *P* for trend |  |  |  | <0.001 | <0.001 |
| Every 1-unit increment |  |  |  | 1.83 (1.72, 1.95) | 1.89 (1.77, 2.02) |
| LAP |  |  |  |  |  |
| Quartile 1 | 1493 | 203 |  | 1.00 | 1.00 |
| Quartile 2 | 1428 | 268 |  | 1.38 (1.13, 1.68) | 1.32 (1.07, 1.62) |
| Quartile 3 | 1370 | 326 |  | 1.75 (1.45, 2.12) | 1.68 (1.36, 2.08) |
| Quartile 4 | 1254 | 442 |  | 2.59 (2.16, 3.12) | 2.49 (2.00, 3.10) |
| *P* for trend |  |  |  | <0.001 | <0.001 |
| Every 1-unit increment |  |  |  | 1.37 (1.29, 1.45) | 1.35 (1.26, 1.45) |
| VAI |  |  |  |  |  |
| Quartile 1 | 1472 | 225 |  | 1.00 | 1.00 |
| Quartile 2 | 1442 | 253 |  | 1.15 (0.95, 1.39) | 1.17 (0.96, 1.43) |
| Quartile 3 | 1372 | 324 |  | 1.54 (1.28, 1.86) | 1.57 (1.29, 1.91) |
| Quartile 4 | 1259 | 437 |  | 2.27 (1.90, 2.72) | 2.35 (1.94, 2.84) |
| *P* for trend |  |  |  | <0.001 | <0.001 |
| Every 1-unit increment |  |  |  | 1.33 (1.26, 1.41) | 1.34 (1.26, 1.43) |

Abbreviations: HTGW, elevated triglyceride level and enlarged waist circumference; HTNW, elevated triglyceride level and normal waist circumference; LAP, lipid accumulation product; NTGW, normal triglyceride level and enlarged waist circumference; NTNW, normal triglyceride level and normal waist circumference; TyG, triglyceride-glucose; VAI, visceral adiposity index.

^*^ N represents sample size for non- diabetes group or for diabetes group.

^†^ Adjustment for age, sex, education, marital status, retirement status, smoking, alcohol drinking, leisure-time physical activity, body mass index, systolic blood pressure, diastolic blood pressure, and history of cardiovascular diseases.
